# State Dental Examining Boards and Their Questions

**Published:** 1900-12

**Authors:** William H. Trueman

**Affiliations:** Philadelphia


### STATE DENTAL EXAMINING BOARDS AND THEIR QUESTIONS.[4]

BY WILLIAM H. TRUEMAN, D.D.S., PHILADELPHIA.

The advent and the evolution of the State Dental Examining Boards has not proved, either to the profession or to the community, wholly good or thoroughly bad. In so far as they were designed to

[4] Read before the Academy of Stomatology, October 23, 1900.

enforce that those who held forth to practise the science and the art of dentistry should prior thereto pursue a suitable course of instruction, they have accomplished much. Unfortunately, however, knowledge and honesty, intelligence and integrity, are not always close associates. While the Boards have the power to enforce a suitable course of instruction, and to demand evidence of knowledge and ability, neither they nor the schools can compel the use of it. The college diploma and the certificate of the State Dental Examining Board may hang side by side in places of business where but few skilful operations are performed and but little honest work done. The disreputable practitioner, since the State Dental Examining Boards have taken in hand to supervise and rerate the college graduate, is able to assert, in his various advertising devices, that not only is every operator in his employ a graduate from one of the best dental colleges in the world, but each one has in addition thereto a certificate endorsing his skill and acquirements as a dental surgeon, issued by the State Dental Examining Board after a rigid and thorough examination. Who can gainsay this assertion? Who of the general public would for a moment hesitate to place himself for dental services, unreservedly, in the hands of one so highly recommended?

The State Dental Examining Boards have thus tended to make disreputable practitioners appear reputable; and instead of protecting members of the community from their wiles, they have been instrumental in throwing dust in their eyes.

The first service rendered the profession by the State Dental Examining Boards was that which tended to discount the private preceptor and lead the profession and the public to a better and more general appreciation of the advantages offered by a thorough, systematic, and well-arranged course of instruction by well-qualified teachers. They led the profession and the public to appreciate the advantages offered by the dental schools well provided with the necessary apparatus and appliances for teaching the practice and theory of dental science in all its bearings. They emphasized, by their examinations, the limited capacity of the preceptor as a teacher, in comparison with a college faculty composed of practitioners, recognized by the profession as skilful and well qualified to teach that branch of dental science they made their chief concern. Regardless of the future, this first service, discrediting the private preceptor's certificate and dignifying the dental college diploma, will ever remain the State Dental Examining Board's most creditable work.

The selection and appointment of examiners was, in Pennsylvania, the duty of the State Dental Society. This Society, then young and vigorous, had in its membership a sufficient number of practitioners to justly consider itself the representative of the profession within the State. The first examining boards had the power to confer upon any applicant meeting their approval, after an examination lasting but a few hours, the same legal rights that otherwise could be acquired only by attendance of many months at a dental college, entailing an expense, for tuition alone, far greater than the trifling examination fee. It was felt to be a matter of serious moment to select for this important office, in justice to the students and the schools and in justice to the community, men of recognized ability and sound judgment, men who were closely in touch and in full sympathy with our educational institutions, in order to insure that their licensees, those who successfully passed their examinations, were, as nearly as could be, considering their lack of training, the equal of a college graduate. It was thought advantageous to have upon the board one or more members of a college faculty, so that they, experienced in teaching and experienced in estimating fitness, could, at critical points, foil those shortcomings from which none closely confined to office practice are free. This also gave to the board tone and character; it inspired confidence; it assisted very materially in bringing to pass the legal requirement of a dental diploma.

The passing of the private preceptor was thought by many to complete the work of the examining boards, and so it did, in so far as the judicious use of the power and confidence reposed in them had, as was intended, impressed on the student that working for a college diploma was better than depending upon the decision of a board adverse to admitting into the profession any who were indisposed to avail themselves of the legitimate means provided therefor.

About this time there was a general waking up in educational matters throughout the world. So general was this revival that all educational concerns, from the kindergarten to the highest schools, classical and professional, felt its impress. It so stirred up our English friends that they were able by a supreme effort to brush aside heretofore unsurmountable obstacles, and so overcome the inertia imposed by a long series of bitter disappointments as to repeat in their own bailiwick, in much the same way, that which we had so successfully accomplished more than a score of years before. Unfortunately, in so doing they laid the foundation for much turmoil in our educational matters, and gave added excuse for the examining boards throughout the land to enter a new *rôle,* a *rôle* presenting many new, complicated, and perplexing problems.

It was in this wise:

In order to make visible distinct to the community the dividing line between those who were and those who were not educationally qualified to enter, or having entered to remain accepted and recognized members of the dental profession, with a right to its designating title, there had been a wise and thoughtful compromise between the schools and the boards. A permanent grading up on the one side and a temporary grading down on the other, in order that eventually the legitimate D.D.S., or its equivalent, should prevail. As was intended, this educational advance (it was an educational advance, the beginning of that which is now so much in evidence) placed the dental profession upon a higher level. This appeared to the public an evidence of marked prosperity. Nothing more natural than that it should be followed by an increasing eagerness to enter the ranks. Preparatory to a still further advance, in order to secure harmonious action the examining boards formed a representative National Association, an example followed a year later by the college faculties, with the same object in view. Primarily, the sole object of each was to mutually assist in a general advance the times called for and opportunity fully warranted. Very early, dissensions arose between these two representative bodies, equally anxious to advance and in perfect harmony regarding the means. The trouble was, which should lead. Between the point of view of a member of a dental examining board and a member of a dental college faculty, as regards this question, there is a vast difference, a difference few stop to consider, and yet it is a difference we must all, in justice, not only consider but respect. Even an ideal member of an examining board assumes, in performing his duties, no personal responsibility. He can, at will, end at any moment his term of office with no pecuniary loss to himself or his fellows; and with but little embarrassment his place can be promptly and acceptably filled. He has no reputation to make or sustain for himself, his colleagues, or his office. No lengthy preparation or study is required, and no expense, save it may be for the purchase of a few quiz books. He is not called upon to sacrifice his present or prospective private practice. The office, when attained, represents merely the favor of his friends or a little effort on his part soliciting votes. Not so with a member of a college faculty. He assumes a personal and pecuniary responsibility. He assumes to sustain the reputation his college has been years in acquiring. He has his own reputation to make in a new *rôle,* and stands shoulder to shoulder with his fellows, sharing with them success or failure and all that success or failure stands for. Not only do his new duties call for long and careful preparation and constant study, but over and above all, they call for a serious sacrifice of private practice, present and prospective. He cannot step in and out of his professorship as jauntily and care-free as can a member of a dental examining board. All this entails legitimate questions of a business character no one has warrant to belittle or to set aside.

For many years, as all veterans in the service too well know, a professorship in a dental college had proved an "expensive luxury." It was eminently wise and proper that those upon whose shoulders the responsibility rested should profit by past experience; should desire to advance slowly; should exercise the caution and discretion of careful, honest business men; and hesitate, until assured that the profession on which they depended for support was ready and willing to follow with substantial aid, before making changes, possibly inviting disaster. Well would it have been had the examining boards, when urging the faculties to extensive and radical changes, taken these matters more into account. Well would it have been had the commercial spirit, which like the trickling ichor from a foul ulcer, corroding and corrupting all it touches, been held in check while this question was being settled.

The profession was gathering itself for a strong and vigorous leap. The public was beginning as never before to appreciate the value of its services in promoting comfort, health, and happiness. It was coming to the fore as a means of livelihood, offering tempting opportunities to those ambitious to accept and receive, on broader lines than those recognized by the slaves of the yardstick and the scale-pan. Men of other and baser mould, others who had missed the golden opportunity which comes but once, and others, again, who had lost through passing years the elasticity of youth, saw nothing in all this but fewer and lessened fees as their fevered imaginations conjured a swarm of competitors around them. They became clamorous for restrictive measures. It was to these a godsend when the General Medical Council of Great Britain announced that the British dental license must be earned in British dental schools. A godsend to the disgruntled here; a welcome godsend to the profession there. It was the making of it. It raised their schools to the same prominence to which ours had been raised by the first dental examining boards. The professional schools are the life, the blood, the manhood of the profession. The profession begins when they begin, grows as they grow, and dies when they die. So thought the General Medical Council of Great Britain.

It was in the air, on the other side, that the American dental college was without a peer; that the British dental schools were of no account. To overturn these prejudices on other than commercial grounds was a delicate task; a task the astute diplomacy of the General Medical Council of Great Britain met and adroitly mastered. Owing to the contentious state of affairs which had long existed, our brethren across the sea were compelled to take with their first breath as a legally recognized profession a most nauseous dose of ignorance and ethical depravity. Taking advantage of the disgust which this naturally created, the Medical Council, having at heart the building up of British dental schools, said, "We have ignorance enough, we will have no more. We close the doors to all uneducated men. Our own schools we can control, they shall demand educated men, and we will recognize no other." It was, under the circumstances, a taking expression, well conceived, and accomplished fully and completely the desired end. It was interpreted on this side as a reflection upon the educational standard of our own dental colleges, and proved to the disgruntled a sweet morsel. To them anything was acceptable; a higher entrance or a sheriff's sale. Anything that promised to restrict the "college output," as they, true to their commercial instincts, termed it. They ranted and raved at the disgrace the low standard of the American colleges had brought upon the heretofore fair name of American dentistry,—whatever that may be; of higher education and higher fees; of the grinding competition of the college clinics; berating the commercialism of dental colleges and dental college professors, until at times their impassioned and frantic appeals for this and that made the dental meeting more like a corner in some ill-conditioned bedlam than a company of professional gentleman discussing their affairs.

One after another the nations of Europe, as a measure to protect "home industries," following the example of Great Britain, refused legal recognition to any but graduates of their own schools. The open door of American dental graduates, of interest to one in a thousand only, was tightly closed. Had those who raised the cry about the discrediting of American dental diplomas been thoughtful readers; had they watched the course of events as portrayed in the journals, they would have known that educational matters contributed to this result, not a feather's weight. American dental colleges, their graduates and diplomas, are credited, respected, and honored the world over to-day as fully and as highly as they ever were. A dentist from the United States is as highly thought of as a dentist, and as warmly welcomed as a professional brother, as ever before. It is only as a competitor that he receives the cold shoulder. The only discrediting the American dental colleges, their graduates and diplomas, have ever received has been in and by the dental societies of the United States. The so-called exclusion has served, however, as many another untruth has served, as a war cry; as an additional excuse for the examining boards to take a fresh start, in a new *rôle,* on a vastly different basis. It was educational; it became commercial. It was conceived to build up, to foster, to widen the field and to give increased importance and value to a well-earned degree. Having accomplished this, in an unguarded moment a mob gained control. It has since essayed to break down, to hamper, to narrow, and belittle the work, the means, and the men their predecessors had labored so earnestly to assist and sustain. Men, educationally incompetent; others, mentally unfitted to form fair and just decisions; and others again, ambitious to be leaders, recklessly aggressive, aping the wiles of social and political agitators, obtained the ascendency. They catered to the drones and the improvident, the incompetent and the indifferent; holding out alluring schemes to secure by legal or repressive measures that which comes only as the reward of earnest, well-directed, persistent efforts. Under this influence the harmony between the educating bodies and the boards was broken. Far more disastrous, however, has been the effect upon the professional societies. Those who looked upon them for post-graduate instruction, or as a means to ripen their experience by comparing and contrasting it with that of others, soon tired of the ill-tempered harangues which displaced scientific discussions; and new-comers were repelled by the constant sneering at colleges from which they had just graduated. It thus came to pass, naturally, that, as the State societies selected the members of the examining boards, the control of the boards passed into the hands of men who had made themselves conspicuous in this unseemly strife, and who sought the office as a vantage ground from which to fight the colleges.

The aggressive policy of the National Association of Dental Examiners reached high-water-mark at the thirteenth annual session, held at Saratoga, beginning August 3, 1896. The official report, published in the *Dental Digest,* vol. ii., 1896, pages 544, 614, 675, and 734, read in connection with the next annual report in the same journal (vol. iii., 1897, pages 512, 588, 696, 751, and 834), is interesting as showing the character and intentions of this organization. At the session of 1896 they first ruled, "That hereafter all boards, members of this Association, accept the list of reputable colleges of the National Association of Dental Examiners as the official list of their respective States." It was understood, although not plainly expressed, that all State boards, members of the National Association, should consider themselves obligated to obtain, as soon as possible, legal recognition of the National body by their respective States. Later, Dr. G. Carleton Brown presented the following resolution, which was passed: "*Resolved,* That colleges recognized by the National Association of Dental Examiners be required to place on their annual announcements and catalogues the fact that they are on the list of colleges recognized by the National Association of Dental Examiners."

At a later stage, the Association adopted a set of "Rules and Conditions for obtaining and maintaining recognition of dental colleges by the National Association of Dental Examiners," in which, as prominently and as forcibly as was its purely educational requirements, this resolution was incorporated as follows: "No college shall be in the list of recognized colleges which does not state in its annual announcements that it complies with the rules and conditions of the National Association of Dental Examiners."

This piece of bombastic folly proved their Waterloo. The colleges very properly refused to recognize an authority that existed only in their inflated imaginations. Could they have succeeded in carrying out the programme here outlined, these doughty leaders might soon have had the dental profession in the United States as thoroughly organized on up-to-date trade union lines as was once the Knights of St. Crispin.

There was no excuse for all this. The aim of these men (apart from their ambition to be considered leaders), set forth plainly in their contributions to the professional journals, was simply and solely to restrict, as a means of reducing competition. The colleges, by far the greater number of them, were managed by men with well-deserved reputations as zealous workers for the public good, men who were carrying on our scientific investigations, devoting, unstintedly, their time, money, and talents to increase the resources of the dental profession. The dental college graduates were being yearly drawn from better men, and were yearly being better educated and trained. The colleges were keeping well to the fore, and were fully meeting the reasonable expectations of the profession.

In proof of this, I point to their crowded classes. In proof that the profession was with the colleges, I draw attention to the fact, too plainly seen to be disputed, that every dental society that has espoused the cause of the element the aggressive examining boards stand for has suffered by it. The great body of the profession has sufficient intelligence to discriminate, unerringly, between the men who work and the men who talk.

I now ask your attention to the questions of the examining boards. By these we are able to judge, fairly well, the mental and educational fitness of the examiners to ascertain and pass upon the ability of the candidate, on other points than those embraced by practical work. The ability to ascertain and judge correctly is the vital point on which the usefulness of examining boards wholly depends. Are they competent to examine and to judge? To so examine and to judge that, on the one side the community is protected (in so far as this provision of dental law is effective) from incompetent practitioners, and on the other side, that no competent candidate is deprived of just and honestly earned rights? To pass an incompetent applicant is a blunder; to reject one competent, and thus deprive the community of valuable services and the applicant of that which means so much, is a crime. On the competency and the honesty of the examining boards depends the vast difference between a useful service and a farce. These examinations can be made of great educational value, or they can be made as ineffective as those of the civil service in the hands of a political machine. They can be made to serve or defeat the best interests of the profession. It all depends upon the men behind the questions. The standard of the examination must be the preamble to the dental law. Its one purpose, to weed out incompetency. Any attempt to subvert this will inevitably lead to disaster. The profession must depend upon itself, not the law, for higher attainments.

I am impressed that the purpose of the examination should be to ascertain the candidate's "stock-in-trade," the provision he has made and has at command to meet the varied requirements of a dental practice. Does he understand? Has he a fair working knowledge of the varied conditions he will be called upon to meet, and of the many expedients the art and science of dentistry provides? Has he a sufficient grasp of the underlying, correlated, and collateral sciences to utilize the assistance they now and again may afford?

My study of this subject has impressed me that dental examining boards fail to consider the importance of this. Very few questions, indeed, are so addressed. Such trivial matters as the comparative usefulness of glycerin or water to temper moulding sand, the right way to use borax, the difference between an atom and a molecule, are repeated time and again, as are immaterial questions directed to office and workroom manipulations that appeal to personal preferences only. As between examining boards of different States, I find but little to choose, and would suggest that if a moiety of the time and thought so fruitlessly spent in considering the interstate license question had been given to a comparative study of examining board questions, much of the turmoil over this matter would have been saved. Beyond the fact that some of their questions contain more words misspelled and incorrectly used, more ungrammatical errors, improperly stated questions, and other blunders (these qualities vary and will continue to vary as the members of the boards change), there is but little to choose between the States that examine. We may admit as substantial claims for preference the differences between the States regarding preliminary requirements and the exaction of a dental college diploma. That which is beyond this and rests upon the real work of an examining board is pharisaical and a mere pretension.

By common consent New York is admitted to have a system of State supervision over educational matters so excellent, so well connected, and so faithfully administered that it is considered a standard. Its dental board has been from the first in excellent hands. It has attended closely to business, taking but little part in dissensions, and has been so open and above-board in all its doings that it well deserves the credit awarded by placing its State in the first rank. It is natural, therefore, to examine its questions first, especially as the official publications of the University of the State of New York, the title under which the State supervising body is known, promptly places us in possession of a full set of questions of every examining body under its charge.[1] The New York Dental Examining Board presents for each subject, at each examination, fifteen questions, requiring answers to ten only. This is an excellent idea, very old and well tested. Not only does it extend the field in which the candidate may prove his ability, but it permits the examiners to suggest new lines of thought to the educators when planning their questions. If it were possible to get upon the examining boards really competent men, men as well qualified for their duties as are those in the college faculties, this would be an exceedingly valuable help in bringing and keeping our educational standards well up to date. So long, however, as the examining boards contain so many inferior men, men blind to their own shortcomings, the faculties will be compelled to occupy the "firing-line" unaided and alone.

With the New York questions, as with most others, those upon subjects other than strictly dental, and on dental subjects somewhat removed from actual practice, questions which may be had "ready-made," selected from the text-books by mere pencilling and allowing the typewriter to do the rest, little or no fault can be found. These sciences change so slowly, and offer so wide a field,

---

[1] These publications are sold at a nominal price. For list and prices, address University of the State of New York, Albany, N. Y., publication department. In addition to the questions, they contain a great deal of useful and reliable information. College Department Bulletin No. 9, February, 1900, Dentistry, is especially valuable, giving as it does a *résumé* of the dental laws, educational requirements, etc., of all the States.

that by simply taking a few pages at a time, with no mental effort a new and equally good set can be formed for each occasion. When, however, the examiner tries his apprentice hand at improving or changing them, either to escape a consciousness of plagiarism, or to serve some special purpose, then there is another story to tell! He usually puts his foot in it. We have an example of this in the question:

"In what way does absorption of food occur?" [1]

In what way, indeed, does absorption of cabbage and beefsteak, pork and beans, "occur"? Occur is a good word to there use. It signifies something that takes place in the nature of an accident; something that comes incidentally, not as the result of design or intention; and, undoubtedly, absorption of food is something far removed from the usual physiological routine. I am somewhat curious to know, however, what answer this examiner has in pickle for the unfortunate dolt who attempts to answer this cunning question.

As we pass to those studies more closely related to dental science, where the examiner feels impelled to originate rather than copy, the poverty of resources, and the extreme mental poverty of the examining board of this, the standard State, is painfully in evidence. The questions themselves are, many of them, trifling. When touching the practical work of the office and laboratory, they are mere reflections of personal opinion and experience. One examiner is an ardent new departurite, and rings it in wherever possible. His favorite, used six times in less than two years,[2] reads as follows:

"In the fusion of the metals for making dental alloys, describe the process used to prevent the volatilization of the tin, zinc, etc."

He recently referred to this, berating the colleges because, although he had, he says, for a score of years taught the profession

---

[1] University of the State of New York, Bulletin No. 14, August, 1897, page 452, question No. 6.

[2] University of the State of New York, Professional Examination papers: 1898, page 241, question No. 12, Examination May 18, 1898; 1898, page 242, question No. 13, examination June 15, 1898; 1899, page 62, question No. 12, examination September 28, 1898; 1899, page 63, question No. 11, examination January 25, 1899; 1899, page 64, question No. 14, examination April 5, 1899; 1900, page 113, question No. 5, examination September 27, 1899.

the right way, more than half of the dental college graduates who came before him either avoided or wrongly answered this question.[1] The vast difference between volatilization and oxidation this learned examiner failed to grasp.

I not only criticise the character of the questions, but that they are so frequently repeated with but insignificant changes. So often is this done, that any one committing to memory the answers to a few sets, knowing nothing more of the subjects embraced, would stand a fair chance of passing the examination. It may naturally be asked, regarding these and other like questions, Why, if they are so simple, do so many students fail? The mental poverty of the examiners, as shown by their questions, is a sufficient answer. They have, to all these stereotyped questions, stereotyped answers. Any answers returned that do not correspond with these, however correct, are, of course, considered wrong. The questions are framed within the narrow field of their own personal opinion and experience, without regard to the text-books or the teaching of the schools. Likewise, the answers.

We have a type of this in the question:[2]

"How should a cavity be treated and filled when the dentine is highly inflamed?"

The question of inflamed dentine is an unsettled one. It is, and has long been, a matter of dispute among our ablest investigators. At present the weight of evidence favors the idea that inflamed dentine is not a correct expression. With so much to choose from, what right has a dental examiner to enforce his own opinions by including in his questions these disputed matters?

I give from the Ohio Dental Examining Board questions of the examination, May, 1900, a sample question. These, published in the *Ohio Dental Journal,* July, 1900, page 344, have been collated, mainly, from those used in New York. It is, I am impressed, a distinct advantage in favor of the examiners for the critic to criticise their published questions; these have, in the publication, passed an editorial supervision, and doubtless their most glaring

---

[1] INTERNATIONAL DENTAL JOURNAL, vol. xx., May, 1899, page 286; Items of Interest, vol. xxii., February, 1900, page 121; Ibid., March, 1900, page 179.

[2] University of the State of New York, Examination Bulletin No. 14, August, 1897, page 467, question 14.

errors have been corrected. The question selected, I presume, is original with the board.

"Give a characteristic action on amalgam in dental alloys, of each, silver, tin, antimony, zinc, and copper."

I frankly admit, I have not yet learned "the characteristic action of silver, etc., on amalgam in dental alloys."

The Maryland Board presents us with two nuts to crack:[1]

"What is meant by metallo-plastic work?

"What is meant by stanno-plastic work?"

Who, besides the examiner, knows? Where did he get these terms?

I have a few choice specimens from those of the Virginia State Board. They are from original papers used at the examination held in June, 1900. The two words, "ætaology" and "groath," are not misspelled; they are good dictionary words if the dictionary is old enough. They may have been taken from one brought over by the Jamestown Fathers.[2] Let me ask, however, Why are these old and uncommon words used in dental examinations? Do they serve any honest purpose in protecting the community from incompetent dentists? Or are they used for the commercial purpose of keeping down competition by misleading some poor fellow more nervous or less perceptive than his brothers?

The question papers before me, from which I copy, were used by a candidate at this examination. I have six only, and am informed that one has been mislaid. They are neatly gotten up, but many of the questions are awkwardly worded; as seems to be the custom more or less with all dental examining boards, as though anything were good enough for a student; for instance:

"Give minute structure of enamel."

The fourth, fifth, and sixth, on histology, are given in this style. They would have been more dignified if the more expressive word "describe" had been used instead of "give."

The second question of this series is more objectionable:

"By what are the teeth retained in their sockets, and give description of it."

---

[1] American Journal of Dental Science, vol. xxxii., July, 1898, page 141.

[2] Groath, for growth, I now and again meet with in works two or three hundred years old. It probably is here by accident or carelessness. Ætiology, and slight variations of it, is still used, mostly by medical writers. Ætaology and aitielogy are unusual. Etiology is by far the better form when used as an English word.

How much better, and how much more explicit, to say, By what are the teeth retained in their sockets? Describe it.

The first question on Operative Dentistry is a stupid one:

"Give treatment of alveola abscess about the point externally."

The third: "Describe the treatment of fungous groath of dental pulp?"

The questions on Prosthetic Dentistry are examples of word jumbling. The first question:

"In examining the mouth for the insertion of an artificial denture, what should be taken into account as to all its conditions and the first two objects to be attained."

The fifth reads:

"How many kinds of plates are used for dentures and upon what do they depend."

Does he refer to the forms of plates, or the material of which they are made? I think it would trouble a Virginia lawyer to find out what this examiner had in view when penning these two questions. There is but one properly worded question in the series, and that is marred by a misspelled word. An examiner who presents at a dental examination such trash would feel very small, indeed, if made to face a witty attorney, and be examined on these questions, in a suit to show cause for rejecting his client.

The fourth question on Materia Medica reads:

"How would you treat the acute poisoning of carbolic acid?"

It will be observed, the wording of the question implies that the acid has been unfortunate; it has been poisoned; and the examiner asks, How should it be treated? If the acid were mine, I would not treat it at all, but discard it. In the first and third questions on Pathology and Therapeutics we find the word "ætaology." While not obsolete, this particular form is so nearly so in dental literature that a well-informed dentist would be quite excusable if he failed to recognize it. The seventh question of this series is another jumble:

"Describe the pathological condition sometimes found analogous to loss of tooth-structure by attrition."

What does he mean? Does he ask for a description of a pathological condition that occasionally resembles attrition, or one resembling attrition at all times, but rarely met with?

I consider it a gross insult to put before a candidate asking the right to practise dentistry a jumble of words like this. I consider it a disgrace to the profession that such incoherent sentences are so often found masquerading as examination questions. It is adding insult to injury to taunt those who fail to understand such stuff as this, by ascribing the failure to their ignorance. In these six question papers, in eighteen instances the interrogation point is either wrongfully used or omitted; in as many more instances, punctuation marks needful to make sense are omitted. I say, needful to make sense—nothing under the sun could make sense of some of these questions.

We will now come nearer home, and consider the questions of our own examining board. I regret to say that we have here nothing to be proud of. It is a very great pity that there is not a more kindly feeling between the educational and examining bodies. I am very sure any one in close touch with the colleges and the instruction there given could have expunged from these questions, at least, their most humiliating features. There seems to be a disposition to use unusual words in place of those in common use, always, of course, incorrectly; so far so in many cases as to obscure the real import of the question, or to render it ambiguous or wholly unintelligible. No care is taken to make the questions clear cut and direct; the words are often jumbled, with no regard to grammatical or euphonious sequence. A common error is in confusing the separate parts of a question by omitting needful punctuation marks, or using an improper connecting word, or uniting into one question words that properly should be formed into two. Take, for instance, the question:[1]

"What is a tendon and its function?"

There is no such thing as "a tendon and its function" known to anatomical science. Why it should be so written passes understanding. What is a tendon? What is its function? is a properly put question. Jumbling the two ideas together indicates either ignorance or design. Another objectionable feature is addressing the questions personally: How would you do thus and so? They would be right and proper so addressed to men of experience; but addressed to men just out of college, not yet admitted to practice, it is absurd. You there want to know what he has in store; you want to know if he understands possible conditions he may have to

---

[1] Report of the Dental Council of Pennsylvania, 1897-98, April, 1898, examination, page 18, Anatomy and Physiology, question No. 2.

meet; and how they may, can, or should be treated. The expert witness style, presenting a supposable case and asking an opinion, is absurd enough in a lawyer's hands, and usually considered there a time-killing device.

In treating a diseased condition, or in outlining a course of treatment, the presence of the patient, the seeing of actual conditions, is far more important than anything spoken or written. There is not a question, proper to be asked, that needs be personally addressed. Another fault is that of addressing questions to *practices* and not to *principles.* Why cannot eighteen carat gold plate be soldered with twenty carat solder? seems, from its frequent repetition, to be a favorite with all examining boards; and what a ridiculous one it is! Make the alloy platinum, and one carat gold plate can be soldered with twenty-four carat solder without the slightest difficulty. If the examiners realized the important work they have in hand, and what it means to pass or reject wrongfully, we would have none of this.

Again, there is thoughtlessness in changing the questions. For instance, December, 1897, the question is asked:

"How are the nutrient products of digestion taken up and carried to the circulation?" [1]

April, 1898, this is thoughtlessly changed so as to make it appear new, and made to read:

"When and how are the products of digestion finally incorporated into the tissues?" regardless of the fact that by far the larger portion of the products of digestion pass through the anus and other excretory channels. The examiner should have known that the fæces, the urine, etc., are as truly products of digestion as is the more nutrient chyle.

There are some questions of doubtful propriety on scientific grounds; for instance:

"In how many and what bones are the teeth located?" [2]

Can the alveolar process, anatomically speaking, be called a bone?

"Explain how it is that heat both oxidizes certain metals and deoxidizes their oxides." [3]

---

[1] Report of the Dental Council of Pennsylvania, 1897-98, pages 15 and 18.

[2] Special Dental Anatomy, December, 1897, question 1.

[3] Chemistry, June, 1898, question 2.

Heat does not oxidize; nothing in the world can oxidize but oxygen.

"Of what class of articulation is that between a tooth and its maxillary, and how is it lubricated?" [1]

Whether the relation of the tooth to its socket warrants the term articulation or not is a disputed point. The examiner evidently thinks it does. But what does he mean by the expression "a tooth and its maxillary"? Maxillary is usually considered an adjective. His suggestion regarding "lubrication" may be a permissible "catch;" but in this decidedly faulty question it is absurd.

"How does tissue outside of the circulations receive its nourishment?" [2]

We have the arterial, venous, capillary, cell to cell, and lymphatic circulations. Outside of these, how can any tissue receive nourishment? I was curious to know what construction the examiner put upon this question, and for that purpose obtained a typewritten transcript of a student's answers. The student was evidently at sea, but amid a few lines of meaningless words had a little to say of capillaries and cells, and the answer was marked ten! I have no doubt but that the candidate understood how tissues were nourished; but, What of his examiner?

"In what parts of the body, and how, is heat generated?" [3]

This question would have been all right half a century ago. It seems based upon the now discarded theories, regarding animal heat, of Lavoisier and his followers.

Some questions are so carelessly written that they fail to convey the idea intended; for instance:

"How would you bring a superior lateral incisor into position that strikes inside the lower teeth?" [4]

The insertion of a comma after position, or, better still, rearranging the words so as to read, How would you bring a superior lateral incisor that strikes inside the lower teeth into position? makes of this faulty question one readily understood.

---

[1] Anatomy, Physiology, and Special Dental Anatomy, April, 1899, question 8.

[2] Ibid., June, 1900, question 6.

[3] Ibid., June, 1900, question 7.

[4] Principles and Practice of Operative and Prosthetic Dentistry, May, 1900, question 7.

"Describe and give the supposed causes for Hutchinsons teeth." [1]

What is here called for? If a description and the cause of Hutchinson's teeth, it should read, Describe Hutchinson's teeth, and give the supposed cause.

"Describe the Kidneys and their functional operation." [2]

How much better to say, Describe the kidneys, and their functions.

"Describe the more minute structures which evolve the sense of taste." [3]

"Evolve" is incorrect. It expresses the idea of growing out of; something coming from something else. It should read, Describe the minute (more minute, if you like) structures in which are located the sense of taste.

"What is meant by the pathology and morbid anatomy of a disease?" [4]

What is meant by pathology and morbid anatomy? is a good question; adding to it "of a disease" makes it absurd.

I find in all dental examination questions, not only of this State, but all that I have examined, an inexcusable carelessness in wording and punctuation. In this respect they are a marked contrast to questions from other examining bodies with which I have compared them; notably those of the Medical Board and those prepared by the school-master for the recent preliminary examinations in this State. The only typographical error in the full series of the last named has been corrected with a pen. All the questions are correct; they have but one meaning; it is a pleasure to read them.

The questions for the May and June (1900) Dental Examinations of this State are distinctly worse than those of previous years, mainly in being marred by incorrect spelling of technical names; for instance, question 9, Materia Medica (June examination), calls for three drugs useful in the treatment of "empyemia of autrum." Other errors of spelling are less misleading, but none the less inexcusable. A large majority of the questions are in need of the school-

---

[1] Dental Pathology and Bacteriology, May, 1900, question 2.

[2] Anatomy, Physiology, and Special Dental Anatomy, June, 1900, question 4.

[3] Dental Histology and Dental Physiology, June, 1900, question 6.

[4] Dental Pathology and Bacteriology, June, 1900, question 4.

master's attention; this is true, however, of all that I have examined as prepared by dental examining boards. The examining board business has been in the past, and is now in some States, conducted in such a star-chamber fashion, by boards, some of them, claiming to be higher than the appointing power, that supervision has been unlooked for; and the work has been done in a slipshod, none-of-your-business sort of a way, as these specimen questions before you plainly show. They are specimens only; they can be duplicated by the score.

I would most earnestly urge upon those elected to serve upon dental examining boards, that when getting their kit of quiz books they add a good modern unabridged dictionary, a good work on English synonymes (that by George Crabb is excellent), and an elementary work on English grammar; and that they make free use of them. They then might avoid such blunders as that in question 2, Anatomy and Physiology, June, 1898,—"What muscles are involved in respiration?" Not a muscle in the human body but what is involved in respiration, although but few are concerned. Better by far stick closely to well-understood, good old-fashioned common words, than to become involved and evolved, induced, seduced, superinduced, and traduced, by a deuced lot of absurd nonsense.

Is it too much to ask that the questions placed before dental students in the examination halls shall be prepared with the same care as would be an article intended for a dental journal? It is no excuse to say that many of the most noticeable blunders are typographical. They may, some of them, have been so; but when they are distributed, uncorrected, to those about to be examined, they then become the questions of the examining board, and the examining board that has accepted and distributed them must shoulder all their errors of every kind and character. *They are then theirs.*

The method of selecting men for this office is faulty. The favorite plan of the State society, nominating two or more, from which number the appointing power ordains one, will never command the best men. It implies that the appointing power is incapable of judging fitness. No man of standing, reputation, and self-respect will willingly permit himself to be a candidate in competition with another, and have his fitness for the position passed upon by a power acknowledged to be incapable of justly deciding. It is objectionable, also, in making unpleasant, on account of calling for the election of a "straw" opponent, the reappointment of an acceptable and well-tried examiner; unless there is a provision, as in New York State, where failure to select a candidate is considered equivalent to expressing a desire that the incumbent be reappointed for another term. It ought, however, to be legally so stated. If the appointing power is capable of judging at all, it should be unhampered. If the responsibility is laid on the State society, its selection should be definite and final. Responsibility should rest somewhere. It is the duty of the State society to require from the boards they either nominate or appoint a report of all their doings, and publish in their annual reports a full list of the questions asked at each examination held, with the name of the examiner responsible for each set. "The board" is a soulless, intangible thing. The society would then know who were and who were not, qualified for the position. As it is now, the societies are compelled to "go it blind." You see the result in the questions placed before you. These are from boards open and honorable enough to make their questions known. You can rest assured that those of the star-chamber concerns are infinitely worse.

Are they the work of illiterates, or trade unionists? Is it carelessness, or cunningness? Are we to accept the authors of these questions as the best men the societies can select, to stand guard at the profession's portals, to protect its educational interests?

Our own board says, in their report,[1] "The board of examiners has been painfully impressed by the lamentably insufficient preliminary education of many of the applicants for examination, as evidenced by the written answers to the questions propounded to them. They not only fail to understand the simplest questions, but are unable adequately to express their answers." Are these questions before you from that same board samples of the simple questions referred to?

Now, gentlemen, in conclusion. What value has an examination conducted by men so careless or illiterate as are the authors of these questions? Have these men been selected for their fitness? Are they the best men the State societies can produce? Are we to accept them as representative of the intelligence, of the educational standing of the societies whose choice they are? How long will the profession tolerate examining boards whose questions invite

---

[1] Report of the Dental Council of Pennsylvania, 1897-98, page 6.

derision and disgust, and who make the profession a laughing-stock for well-informed, educated men?

The time has come to see to it that this important position is filled by competent, up-to-date men; men who know enough to conduct a proper examination, or know enough to know that they do not know, and are willing to merely superintend, leaving the real work in abler hands. If this cannot be done, let the farce end.

